# Right Heart Catheterization: An Antecubital Vein Approach to Reduce Fluoroscopy Time, Radiation Dose, and Guidewires Need

**DOI:** 10.3390/jcm12165382

**Published:** 2023-08-18

**Authors:** Giuseppe Locatelli, Luca Donisi, Luca Mircoli, Federico Colombo, Lucia Barbieri, Gabriele Tumminello, Stefano Carugo, Massimiliano Ruscica, Marco Vicenzi

**Affiliations:** 1 Dyspnea Lab, Department of Clinical Sciences and Community Health, University of Milan, 20122 Milan, Italy; beppe.locatelli41@gmail.com (G.L.); dr.lucadonisi@gmail.com (L.D.); stefano.carugo@unimi.it (S.C.); 2 Department of Cardio-Thoracic-Vascular Diseases, Foundation IRCCS Ca’ Granda Ospedale Maggiore Policlinico, 20154 Milan, Italy; luca.mircoli@policlinico.mi.it (L.M.); federico.colombo@policlinico.mi.it (F.C.); tumminellogabriele@gmail.com (G.T.); 3 Department of Pharmacological and Biomolecular Sciences “Rodolfo Paoletti”, University of Milan, 20133 Milan, Italy

**Keywords:** right heart catheterization, vascular complications, vascular access, antecubital access, fluoroscopy time, radiation exposure, guidewires

## Abstract

Antecubital access for right heart catheterization (RHC) is a widespread technique, even though there is a need to clarify if there are differences and significant advantages compared to proximal vein access. To pursue this issue, we retrospectively identified patients who underwent RHC in our clinic over a 7 year period (between January 2015 and December 2022). We revised demographic, anthropometric, and procedural data, including the fluoroscopy time, the radiation exposure, and the use of guidewires. The presence of any complications was also assessed. In patients with antecubital access, the fluoroscopy time and the radiation exposure were lower compared to proximal vein access (6 vs. 3 min, mean difference of 2 min, CI 95% 1–4 min, *p* < 0.001 and 61 vs. 30 cGy/m^2^, mean difference 64 cGy/m^2^, CI 95% 50–77, *p* < 0.001). The number of patients requiring the use of at least one guidewire was lower in the group undergoing RHC through antecubital access compared to proximal vein access (55% vs. 43%, *p* = 0.01). The feasibility was optimal, as just 0.9% of procedures switched from antecubital to femoral access, with a negligible rate of complications. The choice of the antecubital site exhibits advantages, e.g., a shorter fluoroscopy time, a reduced radiation dose, and a lower average number of guidewires used compared to proximal vein access.

## 1. Introduction

Right heart catheterization (RHC) allows a direct measurement of the right heart chambers’ pressure and indirectly the left filling pressure through the pulmonary arterial wedge pressure. RHC is the gold standard for the invasive assessment of patients with cardiopulmonary hemodynamic disorders (e.g., suspected pulmonary vascular disease, increased left heart filling pressure, or unexplained dyspnea) [1]. Furthermore, several cardiopulmonary disorders (e.g., pulmonary hypertension and left heart disease) require RHC both during the diagnostic rule-out and for optimal clinical management during follow-up or any type of intervention. In this scenario, the clinical setting in which RHC can be performed include intracardiac shunts, valvular heart disease, differentiation between constrictive pericarditis and restrictive cardiomyopathy, heart failure, and assessment of heart or lung transplantation [2,3,4]. Until recently, the strategy of choice for undergoing RHC was through proximal venous access (i.e., subclavian vein, femoral vein, and internal jugular vein) [5,6]. Although RHC is a safe and feasible procedure, vascular damage can rarely occur. The rate of complications tends to be low when performed by experienced operators and is usually limited to problems related to venous access (e.g., ilio-femoral vein thrombosis, femoral pseudoaneurysm, arterio-venous fistula) [7,8]. The feasibility of the radial arterial access during coronary angiograms and the evidence of reducing radiation exposure [9,10] by this approach have shifted attention to the antecubital vein access for RHC, which is now the preferred approach in most centers [6,11]. As for arterial catheterization, the proximal puncture reduces fluoroscopy time without adding complexity or adverse events [5,11].

The present study reports the experience of our reference center for dyspnea and cardiopulmonary diseases, comparing different types of vascular access. We aimed at evaluating the feasibility and safety of the right and left antecubital punctures compared to the proximal vascular approaches and assessing their respective impacts on fluoroscopy time, radiation exposure, and guidewire utilization.

## 2. Materials and Methods

### 2.1. Patients’ Selection

We retrospectively identified patients from the Cath Lab of the Cardiology Department of our institution undergoing RHC from January 2015 to December 2022. We enrolled only patients who performed the RHC at rest or during exercise, therefore excluding those who also underwent diagnostic or therapeutic coronary angiography and any other kind of concomitant invasive procedure. We then revised demographic, anthropometric, and procedural data, including the fluoroscopy time, the radiation exposure, and the total number of additional guidewires used. We also assessed the presence of any complications. BMI has been calculated as weight (Kg)/height (m^2^).

### 2.2. Procedure

During the period under study, RHC was performed by 5 experienced cardiologists (M.V., L.M., F.C., L.B., and G.T.) according to standard technique. Patients were clinically stable, required to fast for at least 12 h before the procedure, and provide written, informed consent for the procedure. Laboratory tests were collected before the procedure to ensure no severe coagulopathy or electrolyte disturbances were present in order to avoid an increased risk of hemorrhage or arrhythmias. Venous access was gained in the operating room with the patient in a supine position. The access site was either through a proximal vein, such as the femoral and internal jugular veins, or through an antecubital vein, such as the cephalic and basilic veins. Site selection was made by the operator considering personal experience and access feasibility. From 2015 to 2020, cannulation of brachial veins was obtained in the Cath Lab by interventional cardiologists using Doppler ultrasonography to identify the most suitable vessel. After 2020, a revised approach was implemented in which, when possible, interventional nurses took the lead in performing the initial venipuncture procedure. This involved the application of a tourniquet, followed by the positioning of a peripheral venous catheter with the support of a 20-gauge needle. Peripheral anesthesia was then achieved with lidocaine infiltration, and the peripheral venous catheter was then exchanged with a 6 French hydrophilic introducer by a cardiologist using the modified Seldinger technique. This implied the default use of a short guidewire in any patient, which was not considered when establishing the total number of guidewires used during the procedure. In the event of a failed approach, the cardiologist selected the antecubital vein and placed the venous access under Doppler ultrasound guidance. The operator used a 6 French hydrophilic introducer via the Seldinger technique. Proximal venous access was pricked by using Doppler ultrasound with the Seldinger technique with a 7 French hydrophilic introducer. A Swan–Ganz catheter was then placed inside the introducer and pushed forward until the right-sided chambers were reached. Once the progression of the catheter was stopped or challenged due to the presence of venous obstructions, anatomical tortuosity, or venous vessels’ spasms, the use of guidewire material was adopted to facilitate the insertion of the catheter. The use of additional guidewires was also possible when, in order to accurately measure pulmonary arterial wedge pressure, the operator wished to cannulate a specific branch of the pulmonary artery other than the one naturally reached by the catheter itself. At the end of the procedure, the introducer was removed, and manual hemostasis was applied for 5–10 min with a sterile dressing placed over the puncture site.

### 2.3. Statistical Analysis

Continuous variables were tested for normality with the Shapiro–Wilk test and expressed as mean ± standard deviation if normally distributed or as median and interquartile ranges if not normally distributed, while categorical variables were expressed as absolute and percentage frequencies. Categorical differences between groups were examined using the chi-squared test. A Mann–Whitney test was used for non-normally distributed variables, while the Student’s *t*-test was used to compare differences between means of normally distributed data. When analyzing the median of more than two groups, the Kruskal–Wallis test, or ANOVA, was used to compare non-normally distributed variables. Data are expressed as the mean (standard deviation) when data are normally distributed and as the median (interquartile range: Q1, Q3) when data are not normally distributed. A *p*-value < 0.05 was considered significant for all statistical determinations. All analyses were performed using IBM SPSS Statistics 28.0 software for Macintosh (SPSS Inc., Chicago, IL, USA).

## 3. Results

**Figure 1:**
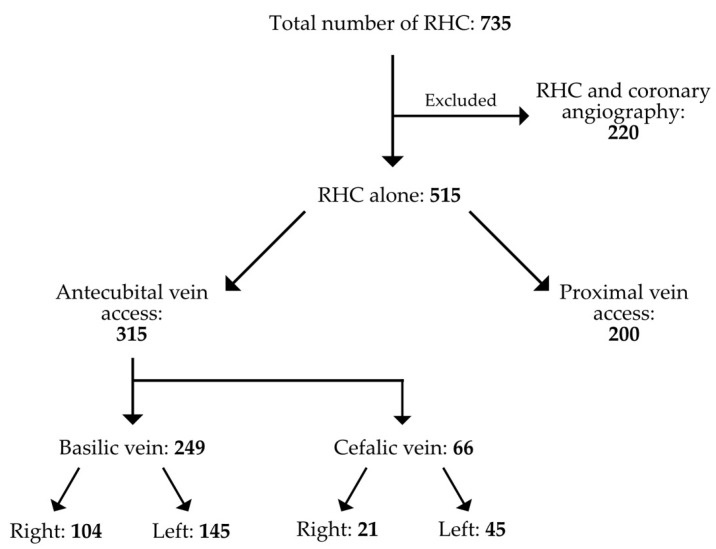
The selection of patients and the frequency of the access site employed.

As represented in Figure 1, a total of 735 patients underwent RHC over an observation period of 7 years.

Of these, 220 were excluded from our retrospective analysis since other invasive procedures (i.e., diagnostic and therapeutic coronary angiography) were performed. The remaining 515 procedures consisted of RHC; 315 were carried out through an antecubital access and 200 through a proximal vein access. These were subdivided into femoral vein access (*n* = 166) and internal jugular vein access (*n* = 34). A single operator performed (M.V.) 272 procedures (52.8%) with a slight preference for antecubital access (72%). In comparison, when considering all operators collectively, the percentage of antecubital access across the board was 61.1%. Data for each single operator are reported in Table S1. RHC through brachial veins was feasible in more than 99% of cases, with only two cases (0.6%) requiring a switch from left to right access and another one (0.3%) in which left antecubital access was switched to femoral access. The rate of complications was negligible, as we had only a single case of late basilic vein thrombosis (within 48 h of the procedure) in the group of patients who underwent RHC through antecubital veins. Furthermore, the feasibility was good, as just three patients (0.9%) eligible for an antecubital access required a crossover either to a new antecubital access or to a femoral access.

In Table 1, it is reported that patients selected for an antecubital access were older (50 ± 21 vs. 57 ± 17 years, *p* < 0.001), and the fluoroscopy time and radiation exposure were lower in this group of patients compared to those who underwent proximal (jugular or femoral vein) vein access (6 vs. 3 min, mean difference of 2 min, CI 95% 1–4, *p* < 0.001 and 61 vs. 30 cGy/m^2^, mean difference 64 cGy/m^2^, CI 95% 50–77, *p* < 0.001). The use of guidewires was significantly reduced in the group of patients with antecubital access compared to proximal vein access (1 vs. 0 with a mean difference of 0.15 [CI 95% 0–0.3], *p* = 0.031), and the number of patients requiring the use of at least one guidewire was lower in the group undergoing RHC through antecubital access compared to proximal vein access (55% vs. 43%, *p* = 0.01). The same results were obtained by further sub-analysis, considering those patients who underwent RHC through proximal vein access, internal jugular vein access, and femoral access. Both groups appeared to manifest increased radiation exposure (112 cGy/m^2^, *p* = 0.003 for internal jugular; 59 cGy/m^2^, *p* < 0.001 for femoral), fluoroscopy time (6 min, *p* = 0.006 for internal jugular; 5 min, *p* < 0.001 for femoral), and guidewire needs (one, *p* = 0.042 for internal jugular; one, *p* = 0.047 for femoral) with respect to patients undergoing antecubital access, as shown in Table S2.

When analyzing the relationship between left (*n* = 190) and right (*n* = 125) antecubital access (Table 2), no difference was observed in radiation exposure, although the fluoroscopy time was significantly reduced in patients having a left-sided approach (3 vs. 4 min, mean difference of 2, CI 95% 1–4, *p* = 0.039). Despite this, the number of patients requiring the use of at least one guidewire was significantly higher in this group (48% vs. 36%, *p* = 0.029). Comparing femoral and jugular access, no statistical difference was found for radiation dose (respectively, median values 59 cGy/m^2^ vs. 112.5 cGy/m^2^, *p* = 0.177), and fluoroscopy time (respectively, median values 5 min vs. 6 min, *p* = 0.707).

## 4. Discussion

Our single-center experience demonstrated that an antecubital vein approach for RHC is a feasible and safe procedure that also reduces fluoroscopy time and radiation exposure when compared to proximal vein access. This evidence is in line with the retrospective study by Shah et al. [11] and the prospective one by Roule et al. [5], who demonstrated safety and feasibility of an antecubital vein approach in patients undergoing RHC while reducing fluoroscopy time. Our results showed at least twice the radiation dose and fluoroscopy time in the case of proximal access compared to antecubital access. This difference is more evident for the radiation dose that reaches its highest value with jugular access.

The result from our already published study partially contrasts with the findings of the REVERE trial [12]. This study was a randomized clinical trial specifically designed to explore any possible difference in terms of radiologic outcomes among patients undergoing cardiac catheterization through a femoral access, a left radial access, or a right radial access. The REVERE trial enrolled 1493 patients randomized in a 1:1:1 fashion and could not show any difference in terms of radiation exposure or fluoroscopy time. According to this observation, we also compared femoral access with an antecubital approach, and a significant difference in radiation dose was confirmed (median value: 59 cGy/m^2^ vs. 30 cGy/m^2^, *p* < 0.001). Indeed, RHC and coronary angiography have different clinical purposes and technical approaches. Thus, it is worth mentioning that the REVERE trial was focused on a population of patients undergoing cardiac catheterization on the arterial side, and anatomic discrepancies existing between the venous and arterial districts may offer a pathophysiologic background to these apparently conflicting results. Moreover, we can speculate that the body thickness, and then the radiation exposure, in the anatomical region (i.e., neck, upper thorax, abdomen) where the venous route flows from proximal puncture to the heart is significantly greater than compared with the antecubital access (i.e., arm, shoulder).

Werner Forssmann was the first to introduce RHC into clinical practice, in 1929. He cannulated the antecubital veins [13,14]. However, the preferred access became the femoral approach until the beginning of the first decade of the new century, when the increasingly utilized radial access for coronary angiography fueled the comeback of the antecubital access. The possibility of access using the small and distal veins of the wrist was also tested but proved to have inferior feasibility with respect to the use of veins located in the antecubital fossa [15]. In our study, the majority of patients underwent RHC through an antecubital vein, and besides receiving a reduced dose of radiation, their procedure required a lower number of guidewires if compared to proximal puncture. This evidence is in contrast with the results of other studies [5,11] reporting that the use of guidewire material was equal within the proximal and antecubital approaches. A possible explanation is that in our center, many patients eligible for RHC suffer from rheumatologic conditions such as systemic sclerosis, thus tending to have smaller and more fragile venous vessels compared to other patients. The presence of such a peculiar population is the reason why, until 2020, any single very proximal antecubital access (e.g., the first third of the brachial vein) was obtained only by very experienced cardiologists under Doppler ultrasound guidance and may justify a lower need for guidewire material to navigate inside the veins of the arm. At the same time, the use of a default strategy using Doppler ultrasound is probably responsible for a higher rate of procedural success with respect to previous experiences [5,11]. Indeed, only a single patient eligible for an antecubital access required a crossover to femoral access, and two patients required a new access on the contralateral side due to the presence of a persistent left superior vena cava. These findings imply a success rate of over 99% for the antecubital approach. Within this scenario, imaging studies performed at the time of clinical follow-up might be useful to select the appropriate technique for RHC and to avoid undesirable anatomical consequences leading to complications. Therefore, we suggest reviewing MRI or CT angiography if they are already available. Conversely, a right antecubital approach should be preferred to limit procedural failure due to anatomical variations. According to expert consensus, the proximal puncture, specifically the right femoral vein, should be chosen when RHC is performed to assess the hemodynamic impact of a large patent foramen ovale or an atrial septal defect [3,6].

Compared to the proximal approach, antecubital access is not only a feasible and safe procedure but also highly versatile. It retains its efficacy and safety even when performing additional examinations, such as exercise RHC to reveal left heart diastolic dysfunction or when measuring the trans-portal gradient in patients suspected of having cirrhotic cardiomyopathy, which is usually performed via the jugular vein [16,17].

**Figure 2:**
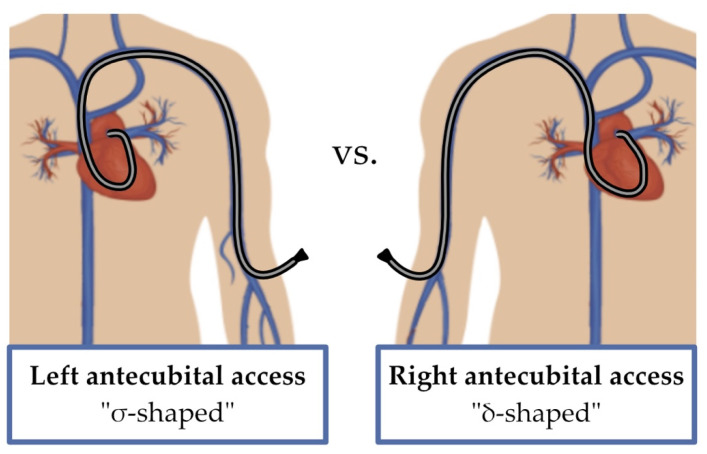
Graphic representation of the σ-shape of the Swan–Ganz catheter with a left antecubital access and the δ-shape with a right antecubital access.

To the best of our knowledge, this is the first time that right and left antecubital access have been compared. Our findings show that patients cannulated on the left side exhibited a statistically significant decrease in fluoroscopy time despite no reduction in radiation exposure. This evidence highlights that left antecubital access requires reduced fluoroscopy time compared to right antecubital access, although no statistically significant reduction in radiation exposure was found. We hypothesized that the presence of anatomical curves inside the major venous vessels up to the right atrium might be responsible for such an unexpected finding. Indeed, we supposed that the σ-shaped (sigma-shape) venous pathway followed by guidewires and catheters in the left antecubital access to reach the right-sided cardiac chambers might be easier to navigate than the δ-shaped (delta-shape) pathway in the right-sided antecubital access (Figure 2).

This could justify why left antecubital access has required reduced fluoroscopy time, but we will need to test it in larger studies in the future. Unexpectedly, we observed a reduced use of guidewire material in patients with a right-sided approach, which may at least partially interfere with the application of the theory mentioned above.

These results should be interpreted within the context of potential limitations. First, our study has a retrospective nature and the intrinsic possibility of selection bias. Any operator’s choice of access was not limited and might be affected by factors such as her/his own expertise and experience with one of the numerous approaches, as well as the patients’ comorbidities. Moreover, one of the operators (M.V.) conducted most of the procedures, and this may have amplified the effect on the results. It is, however, mandatory to observe that the results obtained in the whole population show similar behaviors even when the five operators were considered singularly, especially when it comes to radiation exposure and fluoroscopy time (Table S1). Thus, the advantage of using antecubital access is more evident among experienced operators. Finally, the subjective evaluation of the venous vessels’ status was made by the interventional cardiologist before the procedure, which might have led to an immediate rejection of unfeasible accesses and therefore to an overestimation of the procedural success rate.

## 5. Conclusions

This study confirms that antecubital venous access for RHC is feasible and safe in nearly all patients. The utilization of the antecubital site exhibits advantages such as shorter fluoroscopy time, reduced radiation dose, and a lower average number of guidewires used compared to proximal vein access. While basilic and cephalic veins demonstrated comparable outcomes, left antecubital access showed less fluoroscopy time compared to the right one. These results further support the preferential use of antecubital vein access for basic and complex hemodynamic assessments.

## Figures and Tables

**Table 1:** Baseline and procedural characteristics comparing the proximal and antecubital approaches.

Baseline Characteristics	All (*n* = 515)	Proximal (*n* = 200)	Antecubital (*n* = 315)	Mean Difference or Absolute Difference (95% CI)	*p*-Value
Gender (male, %)	161 (31%)	66 (33%)	95 (30%)		0.497
Age (years)	54 ± 19	50 ± 21	57 ± 17		<0.001
Weight (kg)	61.8 ± 14.1	60.7 ± 14.8	62.6 ± 13.7		0.139
Height (cm)	164 ± 9	163 ± 9	164 ± 9		0.198
BMI (kg/m^2^)	23.0 ± 4.4	22.7 ± 4.5	23.2 ± 4.3		0.268
Radiation dose (cGy/m^2^)	38 (18–79)	61 (28–139)	30 (15–55)	64 (50–77)	<0.001
Fluoroscopy time (min)	4 (2–8)	6 (3–10)	3 (2–6)	2 (1–4)	<0.001
Number of guidewires	0 (0–1)	1 (0–1)	0 (0–1)	0.15 (0–0.3)	0.031
Use of at least 1 guidewire	246 (48%)	109 (55%)	137 (43%)		0.014

Data are represented as mean (standard deviation) for normally distributed variables and as median (interquartile ranges: Q1, Q3) for non-normally distributed variables. CI, confidence interval.

**Table 2:** Baseline and procedural characteristics comparing left to right antecubital accesses.

Baseline Characteristics	Antecubital (*n* = 315)	Left Antecubital (*n* = 190)	Right Antecubital (*n* = 125)	Mean Difference or Absolute Difference (95% CI)	*p*-Value
Gender (male, %)	95 (30%)	67 (35%)	28 (22%)		0.02
Age (years)	57 ± 17	54 ± 17	61 ± 16		<0.001
Weight (kg)	62.6 ± 13.7	63.6 ± 13.8	60.9 ± 13.5		0.093
Height (cm)	164 ± 9	165 ± 9	163 ± 9		0.060
BMI (kg/m^2^)	23.2 ± 4.3	23.3 ± 4.3	22.9 ± 4.2		0.388
Radiation dose (cGy/m^2^)	30 (15–55)	31 (17–49)	31 (15–57)	−2 (4–10)	0.575
Fluoroscopy time (min)	3 (2–6)	3 (2–5)	4 (2–8)	−2 (1–3)	0.039
Number of guidewires	0 (0–1)	0 (0–1)	0 (0–1)	0	0.051
Use of at least 1 guidewire	137 (43%)	92 (48%)	45 (36%)		0.029

Data are represented as mean (standard deviation) for normally distributed variables and as median (interquartile ranges: Q1, Q3) for non-normally distributed variables. CI, confidence interval.

## Data Availability

Data will be available upon request at the corresponding authors’ addresses.

## Supplementary Materials

The following supporting information can be downloaded at: https://www.mdpi.com/article/10.3390/jcm12165382/s1, Table S1: Procedural characteristics for femoral, internal jugular, left antecubital and right ante-cubital venous accesses according to the five operators; Table S2: Baseline and procedural characteristics comparing femoral, internal jugular, and ante-cubital approaches.
