# Supplementary material for: Right Heart Catheterization: An Antecubital Vein Approach to Reduce Fluoroscopy Time, Radiation Dose, and Guidewires Need
Source: J Clin Med. 2023 Aug 18;12(16):5382. doi: 10.3390/jcm12165382 (PMC10456014; doi:10.3390/jcm12165382)
Supplement: Supplementary file 1 [file jcm-12-05382-s001.zip › jcm-2526569-supplementary.pdf]

Table S1. Procedural characteristics for femoral, internal jugular, left antecubital and right antecubital venous accesses according to the five operators.

M.V.	All (n = 272)	Proximal (n = 76) Femoral (n = 56)	Proximal (n = 76) Internal Jugular (n = 20)	Antecubital (n = 196) Left Antecubital (n = 108)	Antecubital (n = 196) Right Antecubital (n = 88)	p-Value
Radiation dose (cGy/m^2)	40 (19–69)	92 (47–195)	118 (27–188)	31 (15–55)	30 (11–57)	<0.001 *
Fluoroscopy time (min)	5 (3–8)	8 (5–12)	6 (2–12)	4 (2–6)	4 (2–8)	<0.001 *
Number of guidewires	0 (0–1)	1 (0–1)	1 (0–1)	0 (0–1)	0 (0–1)	<0.001 *
Use of at least 1 guidewire	127 (46%)	35 (62%)	14 (70%)	53 (49%)	25 (28%)	<0.001 +

L.M.	All (n = 131)	Proximal (n = 71) Femoral (n = 62)	Proximal (n = 71) Internal Jugular (n = 9)	Antecubital (n = 60) Left Antecubital (n = 39)	Antecubital (n = 60) Right Antecubital (n = 21)	p-Value
Radiation dose (cGy/m^2)	43 (22–89)	60 (22–124)	79 (19–120.5)	31 (22–46)	42 (7.5–64)	<0.001 *
Fluoroscopy time (min)	4 (2–7)	5 (3–8)	6 (2.5–8.5)	3 (2–6)	4 (2.5–7.5)	0.057 *
Number of guidewires	0 (0–1)	0 (0–1)	0 (0–1)	1 (0–1)	0 (0–1)	0.456 §
Use of at least 1 guidewire	62 (47%)	28 (45%)	4 (44%)	20 (51%)	10 (47%)	0.853 +

F.C.	All (n = 70)	Proximal (n = 36) Femoral (n = 33)	Proximal (n = 36) Internal Jugular (n = 3)	Antecubital (n = 34) Left Antecubital (n = 27)	Antecubital (n = 34) Right Antecubital (n = 7)	p-Value
Radiation dose (cGy/m^2)	32 (16–62)	37 (18.5–58)	243 (187–398)	27 (14.5–61)	16 (9–33.5)	0.042 §
Fluoroscopy time (min)	3 (2–4)	3 (2–6)	4 (3.5–7.5)	2 (2–4)	3 (2–9)	0.137 §
Number of guidewires	1 (0–1)	0 (0–1)	0 (0–1)	1 (0–1)	1 (0–2)	0.066 §
Use of at least 1 guidewire	37 (53%)	15 (45%)	1 (33%)	16 (59%)	5 (83%)	0.751 +

G.T.	All (n = 22)	Proximal (n = 8) Femoral (n = 4)	Proximal (n = 8) Internal Jugular (n = 4)	Antecubital (n = 14) Left Antecubital (n = 7)	Antecubital (n = 14) Right Antecubital (n = 7)	p-Value
Radiation dose (cGy/m^2)	32 (13–139.5)	97 (22–182.5)	176 (75–241.5)	31 (14–82)	18 (11–35)	0.032 §
Fluoroscopy time (min)	4.5 (2.5–9)	6 (4.5–8)	10 (6–11)	3 (2–5)	3 (2–6)	0.050 §
Number of guidewires	0.5 (0–1)	1 (0.5–1.5)	1 (0.5–1)	0 (0–0)	1 (0–2)	0.098 §
Use of at least 1 guidewire	10 (45%)	4 (100%)	1 (25%)	1 (14%)	4 (56%)	0.762 +

L.B.	All (n = 20)	Proximal (n= 8) Femoral (n = 8)	Proximal (n= 8) Internal Jugular (n = 0)	Antecubital (n = 12) Left Antecubital (n = 9)	Antecubital (n = 12) Right Antecubital (n = 3)	p-Value
Radiation dose (cGy/m^2)	24 (12–44)	33.5 (15–44)		24 (14–61)	23 (7–56)	0.431 *
Fluoroscopy time (min)	4 (2–6)	3 (2–4.5)		4 (5–2)	6 (4–6)	0.155 *
Number of guidewires	1 (0–1)	1 (0.5–1)		0 (0–1)	0 (0–1)	0.143 §
Use of at least 1 guidewire	10 (47%)	6 (75%)		2 (22%)	1 (33%)	0.808 +

p-Value refers to the comparison between proximal and antecubital accesses. Student's T-test was used in the case of normally distributed variables; Kruskal-Wallis was used for non-normally distributed; Chi-Square test was used for non-continuous variables. * Normally distributed variables (Student's T-test). § Non-normally distributed values (Kruskal-Wallis). + Frequencies compared with Chi-Square test.

Table S2. Baseline and procedural characteristics comparing femoral, internal jugular and antecubital approach.

**Baseline Characteristics**	**All (n = 515)**	**Femoral (n = 163)**	**Internal Jugular (n = 36)**	**Antecubital (n = 316)**	**p-Value**
Gender (male, %)	161 (31.3%)	51 (31.3%)	19 (52.8%)	95 (30.1%)	0.245 +
Age (years)	54 ± 19	49 ± 22	57 ± 14	57 ± 17	<0.01 *
Weight (kg)	61.8 ± 14.1	59.6 ± 14.5	65.6 ± 14.4	62.6 ± 13.7	0.027 *
Height (cm)	164 ± 9	163 ± 9	164 ± 10	164 ± 9	0.265 *
BMI (kg/m^2)	23.0 ± 4.4	22.4 ± 4.5	24.2 ± 4.5	23.2 ± 4.3	0.060 *
Radiation dose (cGy/m^2)	38 (18–79)	59 (27–126)	112 (31–189)	30 (15–55)	<0.001 §
Fluoroscopy time (min)	4 (2–8)	5 (3–10)	6 (3–11)	3 (2–6)	<0.001 §
Number of guidewires	0 (0–1)	1 (0–1)	1 (0–1)	0 (0–1)	0.033 §
Use of at least 1 guide-wire	246 (47.8%)	88 (54.0%)	21 (58.3%)	137 (43.4%)	0.030 +

Data are represented as mean (standard deviation) for normally distributed variables and as median (interquartile ranges) for non-normally distributed variables. * Normally distributed variables compared with ANOVA. § Non-normally distributed values compared with Kruskal-Wallis. + Frequencies compared with Chi-Square test.
